# Ecotoxicity Assessment of Nanomaterials: Latest Advances and Prospects

**DOI:** 10.3390/nano14040326

**Published:** 2024-02-07

**Authors:** Vera L. Maria, Angela Barreto

**Affiliations:** Department of Biology & Centre for Environmental and Marine Studies (CESAM), University of Aveiro, 3810-193 Aveiro, Portugal

In the fast-evolving landscape of nanotechnology, the widespread applications of engineered nanomaterials (ENMs) have undoubtedly revolutionized various industries, ranging from healthcare and electronics to agriculture and environmental remediation. As the utilization of ENMs continues to soar, concerns about their environmental impact have become more pronounced [1]. This Special Issue (SI), entitled “Ecotoxicity Assessment of Nanomaterials: Latest Advances and Prospects”, brings together cutting-edge research to explore the intricate world of ENM ecotoxicity. The extensive use of ENMs in over 5367 products (Nanodatabase, January 2023) has led to a surge in their release into the environment, affecting air, water, sediment, and soil [2]. Despite the undeniable benefits of nanotechnology, understanding the potential adverse effects of ENMs remains a significant challenge [3]. The fate, uptake, and biological impact of ENMs are contingent on various factors, such as surface charge, size, shape, and agglomeration state, which are in turn influenced by the surrounding medium [2]. This complexity necessitates the establishment of design rules in nanotechnology research and development to address concerns about environmental and health safety [4].

Key focal topics in assessing the ecotoxicity of nanomaterials [5,6] include:

- Bioavailability, bioaccumulation, and biomagnification: To examine the uptake and distribution of ENMs within organisms, affecting different tissues and organs over time and leading to their accumulation and potential biomagnification across food chains.
- Ecosystem effects: The adoption of a multi-endpoint approach, spanning individual, biochemical, molecular, organismal, and population levels, to provide a holistic understanding of the intricate biological interactions of ENMs.
- Long-term effects: Research with a specific focus on exploring multigenerational and transgenerational responses employing predicted environmental concentrations.
- Standardized testing protocols: To develop and apply consistent testing protocols for assessing the ecotoxicity of ENMs to ensure data comparability.
- Risk assessment: To measure the overall risk of ENMs to the environment, considering exposure levels, toxicity, and potential ecological outcomes.

To date, five interesting papers have been published in this SI, delving into topics such as the ecotoxicity of molybdenum disulfide nanosheets versus its bulk form in soil organisms, the interactions of ENMs with aquatic organisms, the characterization and behavior of silica-engineered nanocontainers in low- and high-ionic-strength media, the influence of the size of boron nitride nanosheets on silkworm development and tissue microstructure, and the effects of combined exposure to carbon nanoparticles and chromium in native and invasive clams. We eagerly welcome additional future studies to further enrich this SI.

As we delve into this SI, we anticipate a wealth of knowledge that will contribute to a more profound understanding of the ecotoxicological profile of nanomaterials. By addressing challenges and proposing future directions, the research presented in this SI will play a pivotal role in shaping the responsible development and deployment of nanomaterials. Through collaborative efforts, the scientific community can pave the way for the sustainable and safe integration of nanotechnology into our daily lives.
